# New Electrochemically-Modified Carbon Paste Inclusion β-Cyclodextrin and Carbon Nanotubes Sensors for Quantification of Dorzolamide Hydrochloride

**DOI:** 10.3390/ijms17122027

**Published:** 2016-12-02

**Authors:** Nawal Ahmad Alarfaj, Maha Farouk El-Tohamy

**Affiliations:** 1 Department of Chemistry, College of Science, King Saud University, P.O. Box 22452, Riyadh 11495, Saudi Arabia; nalarfaj@hotmail.com; 2 General Administration and Medical Affairs, Zagazig University, Zagazig 44519, Egypt

**Keywords:** dorzolamine hydrochloride, potentiometric determination, carbon paste sensor, modified carbon nanotubes sensor, 2-hydroxypropyl β-cyclodextrin

## Abstract

The present article introduces a new approach to fabricate carbon paste sensors, including carbon paste, modified carbon paste inclusion β-cyclodextrin, and carbon nanotubes for the quantification of dorzolamide hydrochloride (DRZ). This study is mainly based on the construction of three different carbon paste sensors by the incorporation of DRZ with phosphotungstic acid (PTA) to form dorzolamide-phosphotungstate (DRZ-PT) as an electroactive material in the presence of the solvent mediator ortho-nitrophenyloctyl ether (*o*-NPOE). The fabricated conventional carbon paste sensor (sensor I), as well as the other modified carbon paste sensors using β-cyclodextrin (sensor II) and carbon nanotubes (sensor III), have been investigated. The sensors displayed Nernstian responses of 55.4 ± 0.6, 56.4 ± 0.4 and 58.1 ± 0.2 mV·decade^−1^ over concentration ranges of 1.0 × 10^−5^–1.0 × 10^−2^, 1.0 × 10^−6^–1.0 × 10^−2^, and 5.0 × 10^−8^–1.0 × 10^−2^ mol·L^−1^ with lower detection limits of 5.0 × 10^−6^, 5.0 × 10^−7^, and 2.5 × 10^−9^ mol·L^−1^ for sensors I, II, and III, respectively. The critical performance of the developed sensors was checked with respect to the effect of various parameters, including pH, selectivity, response time, linear concentration relationship, lifespan, etc. Method validation was applied according to the international conference on harmonisation of technical requirements for registration of pharmaceuticals for human use ICH guidelines. The developed sensors were employed for the determination of DRZ in its bulk and dosage forms, as well as bio-samples. The observed data were statistically analyzed and compared with those obtained from other published methods.

## 1. Introduction

**Figure 1:**
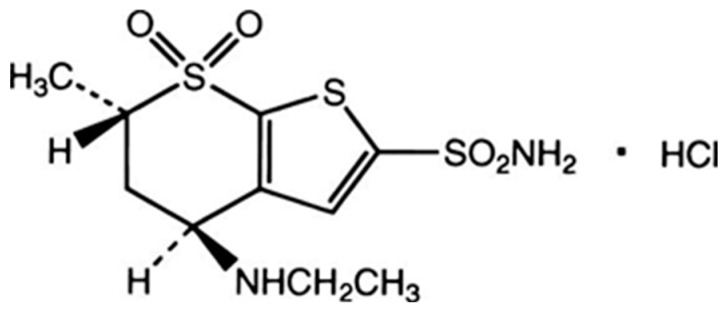
Chemical structure of dorzolamide hydrochloride.

Dorzolamide hydrochloride (DRZ) is a treatment of high pressure inside the eye and helps preventing blindness due to glaucoma. It decreases the aqueous humour due to its nature as a carbonic anhydrase inhibitor [1]. The chemical formula of DRZ is (4S,6S)-4-(ethylamino)-6-methyl-5,6-dihydro-4H-thieno[2,3-b]-thiopyran-2-sulfonamide 7,7-dioxide hydrochloride (Figure 1). The literature survey revealed different analytical methods published for the detection of DRZ, such as spectrophotometry [2,3,4,5] and voltammetry [6]. Other techniques, such as chromatographic separations, were reported for the detection of DRZ in different matrices, including high-performance liquid chromatography [6,7,8,9,10] and thin layer chromatography [11]. With respect to the electrochemical sensors for the detection of DRZ, to the best of our knowledge there is no electrochemical method using potentiometric sensors yet reported.

Carbon paste (CP), is a mixture of pure graphite powder and a pasting liquid used for most laboratory prepared sensors and detectors. The proper function of a sensor in electrochemical analysis is ensured by certain factors, including graphite particle size, its low adsorption capabilities, high chemical purity, and the overall amount of carbon graphite in the carbon paste mixture [12].

Carbon nanotubes (CNTs) are hexagonal networks of carbon atoms which are arranged in the form of a graphite layer rolled up as a cylinder. They are 1 nm in diameter and 1–100 micron in length. There are two types of CNTs, single walled nanotubes (SWNTs) and multi-walled nanotubes (MWNTs) [13]. Due to their extraordinary electrical, mechanical, chemical, and structural properties [14], scientific researchers have converted their diverse research interest from the general characterization of CNT types into more special and interesting studies on their electroanalytical properties. Recently, carbon pastes (CP) and modified carbon nanotube (MCNT) sensors have received great attention. Many applications of these sensors have been published in electrochemical analysis [15], sensors [16], and for medicinal purposes [17].

Cyclodextrins (CDs) are a group of compounds related in structure to cyclic oligosaccharides that have both an external hydrophilic surface and a polar cavity. They have a remarkable ability to form inclusion complexes with various molecules by the insertion of the nonpolar molecule (guest) into the cavity of the CD molecule (host). The normal α, β, and γ-CDs which contain 6, 7, and 8 glucopyranosyl units connected to α-1,4 glycosidic linkages, respectively, have limited aqueous solubility due to the strong intermolecular hydrogen bond in the crystal state. Therefore, the substitution of the hydrogen bond by the hydroxyl group improves their solubility [18].

The literature survey showed many analytical applications, including drug analysis [19], catalysis [20], food analysis [21], and medicine [22].

This study aims to introduce new, simple, highly-sensitive carbon paste sensors for the detection of DRZ. The fabricated sensors are mainly based on the modification of conventional carbon paste sensor with 2-hydroxypropyl β-cyclodextrin and carbon nanotubes. Method parameters were optimized and the critical response characteristics of the fabricated sensors were tested in the selected bulk drug and its ophthalmic solution and bio-samples.

## 2. Results and Discussion

### 2.1. Performance Characteristics of Dorzolamide Carbon Paste Sensors

**Figure 2:**
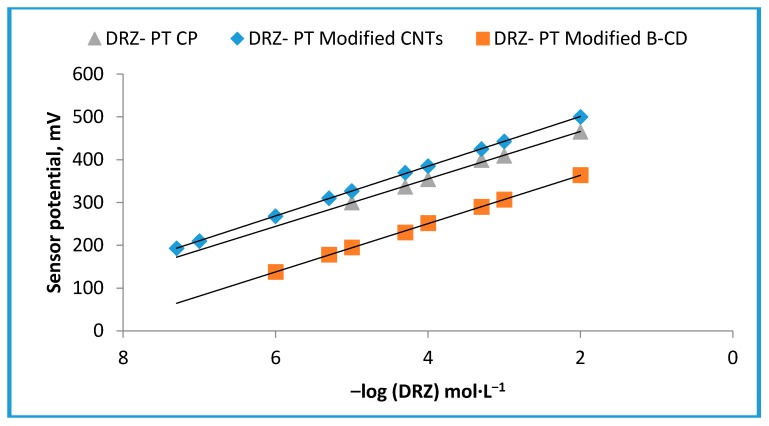
Typical calibration graphs of DRZ-PT carbon paste, modified 2-hydroxypropyl β-CD, and modified CNTs carbon paste sensors.

**Figure 3:**
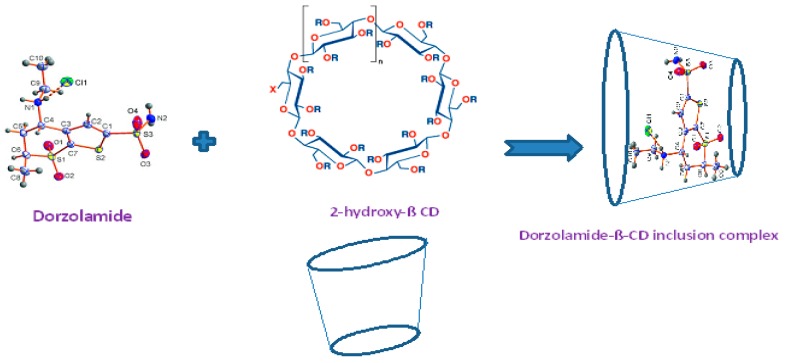
Dorzolamide-β-CD inclusion complex.

The present study introduced three novel fabricated DRZ carbon paste sensors using the electroactive material DRZ-PT, which is readily soluble in organic solvents such as THF. All performance characteristics were studied and are illustrated in Figure 2. The described sensors displayed Nernstian responses of 55.4 ± 0.6, 56.4 ± 0.4 and 58.1 ± 0.2 mV·decade^−1^ over concentration ranges of 1.0 × 10^−5^–1.0 × 10^−2^, 1.0 × 10^−6^–1.0 × 10^−2^, and 5.0 × 10^−8^–1.0 × 10^−2^ mol·L^−1^ with lower detection limits of 5.0 × 10^−6^, 5.0 × 10^−7^, and 2.5 × 10^−9^ mol·L^−1^ for sensors I, II, and III, respectively. The data of critical performance characteristics are presented in Table 1. The chemical structure of β-CD molecule consists of α-1,4 linked α-d-glucopyranose unit with a lipophilic central cavity. Due to the chair formation of the glucopyranose unit with the CD molecule, it appears cone shaped with a secondary OH^−^ group extending from the wider edge and a primary OH^−^ group from the narrow edge. This gives the CD molecules a hydrophilic outer surface. The recorded data in Table 1 revealed that the performance characteristics of the modified β-CD (sensor II) was better than the conventional carbon paste (sensor I) due to the unique characteristic properties of the supermolecular inclusion-CD complex in terms of the high stable complex formed between the cationic drug and the chelating agent of high OH^-^ donation group which is inhibiting the leaching of the electroactive materials from the sensor surface to the test solution and enhancing the electrochemical conductivity that elevates selectivity and sensitivity of the modified β-CD sensor for the determination of DRZ (Figure 3).

Moreover, in the case of modifying sensor II using about 3.0 wt % of CNTs, the performance characteristics were significantly improved due to the chemical stability and good conductivity of the modified CNTs sensor. Additionally, a porous surface structure and large surface area of such sensor facilitated a sensor interface wetting property with solvents [23].

### 2.2. Effect of Plasticizers

The performance characteristics of DRZ-PT carbon paste sensors were greatly influenced by the kind of plasticizer used in the fabrication of these sensors. Therefore, the selection of a suitable plasticizer and the optimization of its amount added to the sensor should be investigated. Four different types of plasticizers, such as di-octylphthalate (DOP) with dielectric constant (ε = 5.1), di-butyl phthalate (DBP) (ε = 6.4), di-butyl sebacate (DBS) (ε = 4.5), di-octyl sebacate (DOS) (ε = 4.0), and *o*-nitrophenyloctyl ether (*o*-NPOE) (ε = 24.0) were added to the carbon paste with various content ratios of (25%, 35%, 45%, 55%, and 65%) and the fabricated sensors were tested. As indicated in Table 2, the most suitable plasticizer was found to be *o*-NPOE. Its suitability can be attributed to the high dielectric constant (ε = 24.0) of *o*-NPOE rather than other types of plasticizers. Additionally, using *o*-NPOE as the plasticizer gave the fabricated sensors highly permeable properties and mechanical stability.

### 2.3. Response Time

The response time is one of the critical parameters which should be evaluated during the detection of selected drugs using carbon paste sensors. With respect to IUPAC recommendations [24] the response time is known as the first instant at which the potential reading of the sensor equals to its steady-state value within 1 mV. Our study was carried out using 1.0 × 10^−8^–1.0 × 10^−2^ mol·L^−1^ of DRZ solution to investigate the response time. The recorded dynamic response time was found to be 60, 45, and 30 s for sensors I, II, and III, respectively, over 45, 65, and 90 days. The fabricated DRZ-PT carbon paste sensors exhibited no significant change in performance characteristics within such periods.

### 2.4. Effect of pH

**Figure 4:**
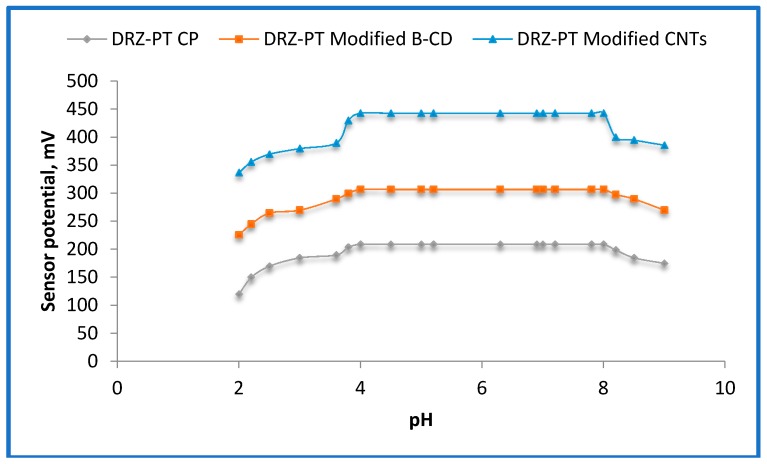
Effect of pH on RZ-PT carbon paste, modified β-CD, and modified CNT carbon paste sensors using 1.0 × 10^−3^ mol·L^−1^ of DRZ solution.

The influence of pH on the fabricated carbon paste DRZ-PT sensors should be studied and optimized. To study the effect of pH on their potential readings 1.0 × 10^−4^ mol·L^−1^ DRZ test solution was firstly acidified using 0.1 mol·L^−1^ hydrochloric acid and then the pH was gradually increased using 0.1 mol·L^−1^ sodium hydroxide. Figure 4 displays the recoded potential readings as a function of −logarithm of the drug concentration. It was found that the safer pH range of the fabricated sensors was at 4–8. The gradual increase of the potential readings for the sensors until reaching pH 4 may be attributed to the increase of the H^+^ in the test solution, while increasing the OH^−^ above pH 8 the potential readings of the sensors were decreased.

### 2.5. Selectivity of Sensors

One of the most important parameters that should be studied is the selectivity of the sensor towards the interested analyte. To our knowledge, the main mechanism of the selectivity was explained by the matching between the locations of lipophilic sites in the two competing species in the bathing solution side and those present in the receptor of ion pairs. To prove the high selectivity of the developed sensors, they were applied for detection of the interested drug in the presence of many possible interferences, such as the common cations, coformulated additives, some sugars, and amino acids. A separate solution method [25] was applied using the following equation:

Log K^pot^_DRZ *J*_*^z+^* = (*E*_2_ − *E*_1_)/S + log(DRZ) − log(*J*^z+^)^1/z^

where, *E*_1_ and *E*_2_ are the sensor potentials in 1.0 × 10^−3^ mol·L^−1^ of both DRZ and the interfering ion *J ^z+^* solution, respectively. *S* is the slope of the calibration plot. As presented in Table 3, the selectivity of the fabricated DRZ-PT carbon paste sensors were evaluated with respect to different interferences, including common cations such as Na^+^, K^+^, NH_4_^+^, Ca^2+^, Mg^2+^, Zn^2+^, Cu^2+^, Fe^3+^, and Al^3+^. Sensor III, which was modified using carbon nanotubes, displayed high selectivity towards the interested drug in the presence of the above-mentioned cations rather than sensor II and sensor I. Furthermore, the addition of β-CD and the formation of a strong supermolecular inclusion complex between the investigated drug and β-CD caused an increase in the sensitivity of the sensor II response rather than that of a conventional CP sensor I and, hence, displayed more selectivity towards the interested analyte. This can be attributed to the high chemical stability of the formed complex, which minimized the leaching of the active material into the bathing solution. In addition, the recorded results revealed that no interferences were displayed by the fabricated sensors in the presence of other compounds, including sugars, amino acids, and the additives.

### 2.6. Effect of Temperature

The potentials of the fabricated DRZ-PT sensors were examined under the effect of different temperature degrees (25, 30, 40, 50 and 60 °C). The isothermal coefficients (dE^0^/dt) of the sensors were evaluated according to the following equation [26]: E^0^ = E^0^ (25) + (dE^0^/dt) (*t*-25), where E^0^ is the standard sensor potential. The recorded data in Table 4 was expressed as the slopes, the standard sensor potential (E^0^) at each temperature, and the usable concentration ranges of the DRZ test solutions. The calculated isothermal coefficients (dE^0^/dt) were found to be 0.000012, 0.000018, and 0.000011 V^0^C^−1^ for sensors I, II, and III, respectively. These results displayed the high thermal stability throughout the investigated temperature ranges.

### 2.7. Effect of Soaking Time

The developed DRZ−PT carbon paste, modified β-CD, and modified CNT sensors were employed to detect the effect of soaking on their performance. First, the optimum immersing time of each fabricated sensor was determined by immersing each sensor separately in 1.0 × 10^−3^ mol·L^−1^ standard DRZ solution. After precondition of the fabricated DRZ-PT sensors the instant response was found to be 12 h for the conventional CP sensor (Sensor I) and approximately 8 h for both modified sensors (Sensor II and sensor III). Excellent Nernstian responses were achieved of 55.4 ± 0.6, 56.4 ± 0.4, and 58.1 ± 0.2 mV·decade^−1^ for sensors I, II, and III, respectively. To investigate the effect of the prolonged soaking of the fabricated sensors on the recorded response, the fabricated sensors were tested upon different intervals of 25, 35, 45, 55, 65, 75, 85, and 90 days. After 35 days a slight decrease of the responses were noticed to be 54.3, 55.6, and 57.8 mV·decade^−1^. Moreover, a sharp inhibition in the potential readings was recorded due to the soaking of the fabricated sensors for 90 days. It was found that after 45 days the potential reading for sensor I was found to be 52.2 mV·decade^−1^, while after 65 and 90 days the potential reading of sensor II and III dropped to 54.4 and 56.7 mV·decade^−1^, respectively. The obtained results showed that the modification of the fabricated CP sensors using β-CD and carbon nanotubes improves the performance characteristics of the sensors. The regeneration of the exhausted sensors was carried out using 1.0 × 10^−2^ mol·L^−1^ of phosphotungstic acid for one day and the same concentration 1.0 × 10^−2^ mol·L^−1^ of the investigated drug for about 8 h. The potential readings of the regenerated sensors were recorded and the obtained data revealed higher Nernastian responses of 54.1, 55.9, and 57.5 mV·decade^−1^ for sensors I, II, and III, respectively. The lifespan of the regenerated sensors was limited to 4, 8, and 12 h for the previously mentioned sensors.

### 2.8. Analytical Applications

#### 2.8.1. Quantification of Dorzolamide Hydrochloride

The developed DRZ−PT carbon paste, modified β-CD, and modified CNT sensors were applied to detect the investigated drug in its bulk powder. As presented in Table 5, the results were calculated as the percent recoveries of 99.1 ± 0.6, 99.3 ± 0.5, and 99.8 ± 0.3 for sensors I, II, and III, respectively. The Student’s *t*-test and *F*-test [27] were employed to analyze the data statistically and compared with those obtained by a published method [4], which is based on the spectrophotometric determination of dorzolamide hydrochloride using potassium bromide in acidic medium in the presence of safranin to produce a pink colour, which absorbs maximally at 540 nm. The proposed sensors were also used to estimate the drug of interest in its dosage forms. The obtained results were 99.2 ± 0.4, 99.6 ± 0.3, and 99.8 ± 0.2 of the three previously mentioned sensors, respectively, as presented in Table 6. Additionally, the suggested DRZ−PT sensors were successfully applied for detection of the selected drug in bio-samples. As summarized in Table 7, the recorded results in human serum were evaluated as 98.9 ± 0.4, 99.1 ± 0.9, and 99.5 ± 0.6 for sensors I, II, and III, respectively, while in human urine the were found to be 98.4 ± 1.2, 99.2 ± 0.5, and 99.7 ± 0.3 for the three fabricated DRZ−PT sensors, respectively.

#### 2.8.2. Content Uniformity Assay of Dorzolamide Ophthalmic Solution

The novel carbon paste sensors of DRZ−PT were applied to determine the content uniformity assay of the drug of interest in its ophthalmic solution. This detection was performed by diluting the content of 10 individual bottles in 100 mL distilled water. The content of each bottle was measured using the proposed sensors and the percent recoveries ± standard deviations were calculated. The results were found to be 99.0 ± 0.6, 99.5 ± 0.2, and 99.6 ± 0.1 for the three described sensors, respectively.

### 2.9. Method Validation

The international conference on harmonization of technical requirements for registration of pharmaceuticals for human use ICH guidelines [28] were applied to ensure the suitability of the proposed method. Method validation was investigated with respect to various analytical parameters, including linearity, accuracy and precision, robustness, ruggedness, etc.

#### 2.9.1. Linearity and Lower Limit of Detection

The linear relationship of the fabricated DRZ−PT carbon paste, modified β-CD, and CNT sensors were investigated using the drug concentration range of 1.0 × 10^−8^–1.0 × 10^−1^ mol·L^−1^. The linear concentration ranges of 1.0 × 10^−5^–1.0 × 10^−2^, 1.0 × 10^−6^–1.0 × 10^−2^, and 5.0 × 10^−8^–1.0 × 10^−2^ mol·L^−1^ for the sensors I, II, and III, respectively were recorded. An increase in sensitivity of the modified CP sensor using 2-hydroxypropyl-β-CD than the conventional carbon paste sensor was observed. While, due to the porous large surface area and the best sensor electrolyte interface of the modified CNT sensor, we can notice the higher performance characteristics of sensor III than sensors I and II. The evaluated lower detection limits were found to be 5.0 × 10^−6^, 5.0 × 10^−7^, and 1.9 × 10^−8^ mol·L^−1^ for sensors I, II, and III, respectively.

#### 2.9.2. Accuracy and Precision

The standard addition method was employed to ensure the accuracy of the introduced method. The recorded data were calculated as percentage recoveries and they were found to be 99.2 ± 0.9, 99.7 ± 0.6, and 99.8 ± 0.1 for sensors I, II, and III, respectively. The precision of the proposed method was ensured using intra-day and inter−day assay. The precision test was carried out using nine replicates of the tested drug solution and the calculated %RSD values were recorded as 0.9%, 0.4%, and 0.1% for intra-day assay and 0.7%, 0.5%, and 0.2% for inter-day detection of DRZ in Dorzolamide^®^ ophthalmic solution using DRZ-PT sensors. The evaluated results indicated good precision with %RSD, which was less than 2%.

#### 2.9.3. Robustness and Ruggedness

The robustness of the developed carbon paste DRZ−PT sensors were examined by introducing a small change in method parameters such as pH value 8 ± 1 using phosphate buffer. The results were calculated as percentage recoveries ± standard deviations. The robustness of the three carbon paste DRZ−PT sensors were found to be 98.7 ± 0.5, 99.2 ± 0.5, and 99.4 ± 0.2 for sensors I, II, and III, respectively. Furthermore, the ruggedness of the developed method was evaluated using another pH meter (Jenway 3310). The calculated percentage recoveries were found to be 98.9 ± 0.8, 99.3 ± 0.4, and 99.8 ± 0.1 for the three mentioned sensors, respectively, revealing no significant differences with those recorded from standard drug solutions.

## 3. Experimental

### 3.1. Materials and Reagents

Pure grade of dorzolamide hydrochloride was supplied by Tokyo Chemical Industry Co. (Tokyo, Japan). Trusopt^®^ 2% dorzolamide hydrochloride, 5 mL ophthalmic solution was obtained from local drug stores. Phosphotungstic acid 99.9%, multi-wall carbon nanotubes powder (carbon > 95.0%, O.D. × L 6–9 nm × 5 µm), graphite powder (1–2 µm), 2−hydroxypropyl β-cyclodextrin (β-CD) and high molecular weight of polyvinyl chloride (PVC) were purchased from Sigma-Aldrich (Hamburg, Germany). Potassium monobasic phosphate, potassium dibasic phosphate and zinc sulfate ≥99.0% were purchased from WinLab Suppliers (East Midlands, UK). Plasticizers of analytical grade were supplied by Fluka (Gallen, Switzerland) such as, *o*-nitrophenyloctyl ether (*o*-NPOE) 99.0%, di-butyl sebacate (DBS) >97.0%, di-butyl phthalate (DBP) 99.0%, di-octyl sebacate (DOS) ≥97.0%, and di-octylphthalate (DOP) 99.5%, Additionally, tetrahydrofuran (THF) as well as hydrochloric acid 36.5%, acetonitrile, and sodium hydroxide pellets 98.0% were purchased from BDH Laboratory Supplies (Philadelphia, PA, USA). Commercial serum samples were purchased from Multi-Serum Normal, Randox Laboratories (Crumlin, Antrim, UK). Healthy volunteers provided the urine samples, informed consent was obtained from all of them prior to the start of the study, and the medical ethics committee in the College of Medicine, King Saud University, approved this study.

### 3.2. Instruments

All potentiometric and pH detection was performed using a HANNA instruments model-211 microprocessor pH-meter (Cluj, Romania). The temperature of test solution was controlled using an AREX heating magnetic stirrer connected to a circulator thermostat. The external reference electrode connected to the system was an Ag/AgCl electrode. A scanning electron microscope (SEM), JEOL JSM-6060 LV (Akishima, Tokyo, Japan), was employed to examine the surface structure of the carbon nanotubes sensor. An Ivyman distiller system AC-L8 was used for providing distilled water.

### 3.3. Preparation of Analytical Solutions

#### 3.3.1. Standard Drug Solution

A standard solution of 1.0 × 10^−1^ mol·L^−1^ DRZ was prepared by dissolving 0.4 g in 10 mL distilled water. The proposed method was applied using working solutions in the range of 1.0 × 10^−8^–1.0 × 10^−1^ mol·L^−1^ which was prepared by serial dilutions using distilled water.

#### 3.3.2. Preparation of Dorzolamide Hydrochloride Ophthalmic Solution

The content of five bottles of Trusopt^®^ 2% dorzolamide hydrochloride (5 mL) was mixed well. Accurate amounts of DRZ solution equivalent to prepared molar concentrations of DRZ solutions of 1.0 × 10^−5^–1.0 × 10^−2^, 1.0 × 10^−6^–1.0 × 10^−2^ and 5.0 × 10^−8^–1.0 × 10^−1^ mol·L^−1^ was diluted with distilled water for sensors I, II, and III, respectively.

#### 3.3.3. Preparation of Human Serum and Urine Samples

Biological samples, such as human serum and urine, were employed to detect the selected drug DRZ using the three novel fabricated carbon paste sensors. The spiking technique was applied and the pH of the bio-samples were adjusted to pH 6 using phosphate buffer. The previously adjusted samples were spiked with an accurate amount of the tested drug. About 1.0 mL of spiked human serum was firstly deproteinated by adding 1.0 mL of acetonitrile, 0.1 mL of NaOH (0.1 mol·L^−1^), and followed by 1.0 mL of ZnSO_4_·7H_2_O (5.0% *w*/*v*). Then the sample was centrifuged at 3500 rpm for 30 min and filtered using a 0.45 milli-pores membrane filter. Human urine was diluted with distilled water and the prepared samples were subjected to analysis without further treatment.

### 3.4. Preparation of Dorzolamide-Phosphotungstate Ion Pairs

The electroactive material DRZ-PT was prepared by mixing 50 mL of 1.0 × 10^−2^ mol·L^−1^ DRZ solution with 50 mL of phosphotungstic acid (PTA) solution. The obtained precipitate was filtered, washed with distilled water, and then left aside to dry for one day at 25 °C temperature. Elemental analysis was performed to confirm the prepared electroactive material DRZ-PT with respect to the percent content of C, H, and N. The recorded data revealed that the formed ion-pair was [C_10_H_17_ClN_2_O_4_S_3_]_3_[PW_12_O_40_] which indicated that the formed ion pair DRZ:PT was found to be 1:1 and the percentages of H, C, and N were theoretically calculated to be 1.30%, 9.10%, and 2.12%, respectively. The found percentages of the previously mentioned elements were 1.29%, 9.12%, and 2.24% for H, C, and N, respectively.

### 3.5. Sensor Fabrication

#### 3.5.1. Fabrication of the Carbon Paste Sensor

A carbon paste sensor was simply fabricated using about 60% of pure graphite powder (1–2 μm), 10.0% DRZ-PT as the electroactive material, and 30.0% *o*-NPOE as a liquid plasticizer. The above contents were mixed well to obtain a homogenous paste and carefully packed in a Teflon holder (3.0 mm in diameter). The connection was carried out using a copper rod. The surface of the sensor was polished to be shiny using tissue paper before being used for potentiometric detections. The sensor was preconditioned in 1.0 × 10^−3^mol·L^−1^ DRZ solution for 12 h for the carbon paste sensor and 8 h for both modified sensors. The cell assembly for all potentiometric measurements was carbon paste/test solution/Ag/AgCl reference electrode.

#### 3.5.2. Preparation of the Modified Carbon Nanotubes Sensor

**Figure 5:**
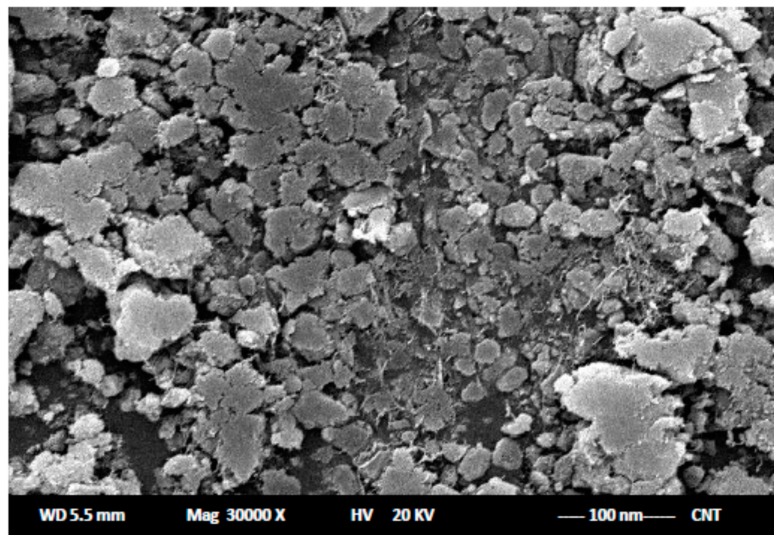
The surface structure of carbon paste sensors using SEM.

To prepare the modified carbon nanotubes sensor, a small amount, about 3%, of multi-wall carbon nanotube particles was added to 57% of pure graphite powder (1–2 μm), 10.0% DRZ-PT as the electroactive material, and 30.0% *o*-NPOE as a liquid plasticizer. The paste was homogeneously mixed, then the procedure, as previously mentioned, was applied. As seen in Figure 5, the surface of the carbon paste sensors were investigated under a scanning electron microscope (SEM).

#### 3.5.3. Preparation of the Modified β-Cyclodextrin Carbon Paste Sensor

The modification of β-CD carbon paste sensor was achieved by adding about 10% of 2-hydroxypropyl β-CD to 50% of pure graphite powder (1–2 μm), 10.0% DRZ-PT as the electroactive material, and 30.0% *o*-NPOE as the liquid plasticizer. Additionally, the previously mentioned procedure was followed.

### 3.6. Sensor Calibration

To construct the sensor calibration graphs all potential readings were plotted as a function of the –logarithm of drug concentrations by applying DRZ-PT carbon paste sensors. Working solutions containing 1.0 × 10^−5^–1.0 × 10^−2^, 1.0 × 10^−6^–1.0 × 10^−2^, and 5.0 × 10^−8^–1.0 × 10^−1^ mol·L^−1^ of DRZ were applied for sensors I, II, and III, respectively.

### 3.7. Standard Addition Method

The standard addition method [24] was applied to detect the selected DRZ drug in its pharmaceutical formulations. Small additions of DRZ solution were used and the potential readings of each constructed sensor were recorded. To detect the concentration of the tested sample, the following equation was applied: (Δ*E* = *E*_2_ − *E*_1_), where, *E*_1_ is the sensor potential of the sample and *E*_2_ is the sensor potential after adding the drug. The change in potential can be used to detect the concentration of the tested sample.

## 4. Conclusions

In the introduced study, three novel sensors for the determination of DRZ were fabricated by the incorporation of DRZ with phosphotungstic acid to produce DRZ-PT as electroactive material. The three fabricated sensors were applied as a conventional carbon paste (sensor I), while sensors II and III were modified using β−cyclodextrin and carbon nanotubes, respectively. Excellent Nernstian responses 55.4 ± 0.6, 56.4 ± 0.4, and 58.1 ± 0.2 mV·decade^−1^ over concentration ranges of 1.0 × 10^−5^–1.0 × 10^−2^, 1.0 × 10^−6^–1.0 × 10^−2^, and 5.0 × 10^−8^–1.0 × 10^−2^ mol·L^−1^ with lower detection limits of 5.0 × 10^−6^, 5.0 × 10^−7^, and 2.5 × 10^−9^ mol·L^−1^ for sensors I, II, and III, respectively, were observed. The developed sensors were employed to determine the selected drug in bulk powder, its ophthalmic solution and bio-samples such as serum and urine. The obtained results indicated that the sensitivity and selectivity of sensor II was improved than that of sensor I due to the addition of β-cyclodextrin as a modifier, while, significant sensitivity for the detection of the interested drug was observed by adding carbon nanotubes to sensor III. The obtained data were treated statistically and displayed significant agreement with those obtained from the published methods.

## Figures and Tables

**Table 1:** Electrochemical response characteristics of DRZ-PT carbon paste, modified β-cyclodextrin, and modified carbon nanotubes carbon paste sensors.

Parameter ^a^	DRZ-PT Carbon Paste (Sensor I)	DRZ-PT Modified β-CD (Sensor II)	DRZ-PT Modified CNTs (Sensor III)
Slope (mV·decade^−1^)	55.4 ± 0.6	56.4 ± 0.4	58.1 ± 0.2
Intercept	376.9	476.8	616.8
Regression equation	E_mV_ = (55.4 ± 0.6) log(DRZ)+ 376.9	E_mV_ = (56.4 ± 0.4) log(DRZ) + 476.77	E_mV_ = (58.1 ± 0.2) log(DRZ) + 616.8
Correlation coefficient, *r*	0.9991	0.9997	0.9999
Linear range (mol·L^−1^)	1.0 × 10^−5^–1.0 × 10^−2^	1.0 × 10^−6^–1.0 × 10^−2^	5.0 × 10^−8^–1.0 × 10^−2^
LOD	5.0 × 10^−6^	5.0 × 10^−7^	2.5 × 10^−9^
Response time/s	60	45	30
Working pH range	4–8	4–8	4–8
Lifetime/day	45	65	90
Temperature °C	25 °C	25 °C	25 °C
Accuracy (%)	99.2 ± 0.9	99.7 ± 0.6	99.8 ± 0.1
Robustness ^b^	98.7 ± 0.5	99.2 ± 0.5	99.4 ± 0.2
Ruggedness ^c^	98.9 ± 0.8	99.3 ± 0.4	99.8 ± 0.1

^a^ Mean of six measurements; ^b^ A small variation in method parameters was carried out at the pH of phosphate buffer (pH 8.0 ± 1); ^c^ Comparing the results with those obtained by different sensor assemblies using a (Jenway 3510, Staffordshire, UK) pH meter.

**Table 2:** Effect of plasticizers on the slopes of the constructed DRZ-PT sensors.

Plasticizer	DRZ-PT CP (Sensor I)	DRZ-PT Modified β-CD (Sensor II)	DRZ-PT MCNTs (Sensor III)
DOS	46.3	51.4	52.9
DBS	50.9	53.7	53.1
DBP	53.5	55.9	56.7
DOP	54.9	56.0	57.9
*o*-NPOE	55.2 *	56.4 *	58.1 *

* The optimum value for the studied sensors.

**Table 3:** Selectivity coefficients (K^pot^_DRZ_^+^) for DRZ-PT sensors using a separate solution method (1.0 × 10^−3^ mol·L^−1^ dorzolamide hydrochloride).

Interferent	K^Pot^_DRZ_^+^		
	DRZ-PT CP (Sensor I)	DRZ-PT modified β-CD (Sensor II)	DRZ-PT MCNTs (Sensor III)
Na^+^	2.5 × 10^−3^	4.1 × 10^−3^	1.2 × 10^−5^
K^+^	3.1 × 10^−3^	3.3 × 10^−4^	7.5 × 10^−4^
NH^4+^	4.5 × 10^−3^	5.5 × 10^−4^	3.5 × 10^−5^
Ca^2+^	5.9 × 10^−4^	6.2 × 10^−4^	5.6 × 10^−6^
Mg^2+^	8.2 × 10^−4^	7.2 × 10^−5^	1.6 × 10^−5^
Zn^2+^	5.6 × 10^−4^	6.3 × 10^−4^	6.9 × 10^−4^
Cu^2+^	1.8 × 10^−3^	4.2 × 10^−4^	5.8 × 10^−4^
Fe^3+^	8.3 × 10^−4^	1.2 × 10^−4^	4.9 × 10^−5^
Al^3+^	4.1 × 10^−3^	6.4 × 10^−4^	7.8 × 10^−5^
Glucose	2.6 × 10^−5^	1.0 × 10^−4^	3.6 × 10^−5^
Lactose	5.6 × 10^−4^	2.8 × 10^−4^	6.9 × 10^−5^
Maltose	5.5 × 10^−5^	1.3 × 10^−5^	5.2 × 10^−6^
Starch	1.4 × 10^−5^	7.1 × 10^−4^	6.3 × 10^−4^
Alanine	4.9 × 10^−5^	9.9 × 10^−5^	8.6 × 10^−5^
Glycine	3.9 × 10^−4^	5.5 × 10^−5^	3.8 × 10^−5^
Histadine	9.0 × 10^−5^	2.2 × 10^−4^	2.4 × 10^−5^
Leucine	4.8 × 10^−4^	3.8 × 10^−4^	8.9 × 10^−6^
Ornithine	5.7 × 10^−4^	6.7 × 10^−5^	5.8 × 10^−5^
Glutamine	9.3 × 10^−4^	9.8 × 10^−4^	2.8 × 10^−5^

**Table 4:** Performance characteristics of DRZ-PT sensors at different temperatures.

Type of Sensors	Temperature (°C)	Slope (mV decade^−1^)	Usable Concentration Range	E^0^ (mV) ^a^
DRZ-PT carbon paste (Sensor I)	25	55.2 *	1.0 × 10^−5^–1.0 × 10^−2^	100
	30	56.4	5.0 × 10^−5^–1.0 × 10^−3^	109
	40	57.6	1.0 × 10^−5^–1.0 × 10^−3^	117
	50	57.9	1.0 × 10^−4^–1.0 × 10^−4^	120
	60	59.0	5.0 × 10^−4^–1.0 × 10^−4^	128
DRZ-PT modified β-CD carbon paste (Sensor II)	25	56.4 *	1.0 × 10^−6^–1.0 × 10^−2^	250
	30	56.8	1.0 × 10^−5^–1.0 × 10^−2^	264
	40	58.2	1.0 × 10^−5^–1.0 × 10^−3^	273
	50	59.5	5.0 × 10^−5^–1.0 × 10^−3^	280
	60	61.4	5.0 × 10^−5^–1.0 × 10^−4^	291
DRZ−PT modified CNTs (Sensor III)	25	58.1 *	5.0 × 10^−8^–1.0 × 10^−2^	475
	30	59.5	5.0 × 10^−7^–1.0 × 10^−3^	481
	40	60.2	5.0 × 10^−7^–1.0 × 10^−3^	487
	50	61.7	5.0 × 10^−6^–1.0 × 10^−4^	493
	60	63.4	5.0 × 10^−5^–1.0 × 10^−4^	501

^a^ E^0^: Standard sensor potential against normal hydrogen electrode (NHE); * The selected slope for each sensor.

**Table 5:** Analytical results for the determination of dorzolamide hydrochloride in its pure form using DRZ-PT carbon paste, modified β-cyclodextrin, and modified carbon nanotube carbon paste sensors.

Samples	DRZ-PT CP (Sensor I)			DRZ-PT Modified β-CD (Sensor II)			DRZ-PT MCNTs (Sensor III)			Recovery (%) (Reference Method [4])
	Taken −log conc. (mol·L^−1^)	Found –log conc. (mol·L^−1^)	Recovery (%)	Taken −log conc. (mol·L^−1^)	Found –log conc. (mol·L^−1^)	Recovery (%)	Taken −log conc. (mol·L^−1^)	Found −log conc. (mol·L^−1^)	Recovery (%)	
Pure drug	5.0	4.97	99.4	6.0	5.99	99.8	7.3	7.28	99.7	
	4.3	4.25	98.8	5.3	5.28	99.6	7.0	6.99	99.9	
	4.0	3.96	99.0	5.0	4.99	99.8	6.0	6.00	100.0	
	3.3	3.28	99.4	4.0	3.97	99.3	5.0	5.00	100.0	
	3.0	2.99	99.7	3.0	2.95	98.3	4.0	3.99	99.8	
	2.0	1.96	98.0	2.0	1.99	99.5	3.0	2.98	99.3	
							2.0	2.00	100.0	
Mean ± SD	99.1 ± 0.6			99.3 ± 0.5			99.8 ± 0.3			99.6 ± 0.4
*n*	6			6			7			6
Variance	0.36			0.25			0.09			0.16
%SE **	0.24			0.32			0.11			0.16
*t*-test	1.733 (2.228) *			0.839 (2.228) *			1.030 (2.201) *			
*F*-test	2.25 (5.05) *			1.56 (5.05) *			1.78 (4.93) *			

* Figures in parentheses are the tabulated values of *t*- and *F*-tests at 95% confidence limit [27]; ** %SE = SD/√*n*.

**Table 6:** Analytical results for the determination of dorzolamide hydrochloride in a pharmaceutical preparation using DRZ-PT carbon paste, modified β-cyclodextrin, and modified carbon nanotube carbon paste sensors.

Samples	DRZ-PT CP (Sensor I)			DRZ-PT Modified β-CD (Sensor II)			DRZ-PT MCNTs (Sensor III)			Recovery (%) (Reference Method [4])
	Taken −log conc. (mol·L^−1^)	Found −log conc. (mol·L^−1^)	Recovery (%)	Taken −log conc. (mol·L^−1^)	Found −log conc. (mol·L^−1^)	Recovery (%)	Taken −log conc. (mol·L^−1^)	Found −log conc. (mol·L^−1^)	Recovery (%)	
DRZ-solution^®^ 22.3 mg·mL^−1^	5.0	4.96	99.2	6.0	6.00	100.0	7.3	7.29	99.9	
	4.3	4.28	99.5	5.3	5.26	99.2	7.0	7.00	100.0	
	4.0	3.99	99.8	5.0	4.96	99.2	6.0	5.99	99.8	
	3.3	3.27	99.1	4.0	4.00	100.0	5.0	4.98	99.6	
	3.0	2.98	99.3	3.0	2.99	99.7	4.0	4.00	100.0	
	2.0	1.97	98.5	2.0	1.99	99.5	3.0	3.00	100.0	
							2.0	1.99	99.5	
Mean ± SD	99.2 ± 0.4			99.6 ± 0.3			99.8 ± 0.2			99.5 ± 0.4
*n*	6			6			7			6
Variance	0.16			0.09			0.04			0.16
%SE **	0.16			0.12			0.08			0.16
*t*-test	1.326 (2.228) *			0.500 (2.228) *			1.677 (2.201) *			
*F*-test	1.00 (5.05) *			1.78 (5.05) *			4.00 (4.93) *			

* Figures in parentheses are the tabulated values of *t*-and *F*-tests at 95% confidence limit [27]; ** %SE = SD/√*n*.

**Table 7:** Analytical results for the determination of dorzolamide hydrochloride in bio-samples using DRZ-PT carbon paste, modified β-cyclodextrin, and modified carbon nanotube carbon paste sensors.

Samples	DRZ-PT CP (Sensor I)			DRZ-PT Modified β-CD (Sensor II)			DRZ-PT MCNTs (Sensor III)		
	Taken −log conc. (mol·L^−1^)	Found −log conc. (mol·L^−1^)	Recovery (%)	Taken −log conc. (mol·L^−1^)	Found −log conc. (mol·L^−1^)	Recovery (%)	Taken −log conc. (mol·L^−1^)	Found −log conc. (mol·L^−1^)	Recovery (%)
Human serum	5.0	4.95	99.0	6.0	5.99	99.8	7.3	7.29	99.9
	4.3	4.25	98.8	5.3	5.26	99.2	7.0	6.96	99.4
	4.0	3.96	99.0	5.0	4.97	99.4	6.0	6.00	100.0
	3.3	3.26	98.8	4.0	3.98	99.5	5.0	4.99	99.8
	3.0	2.99	99.7	3.0	2.92	97.3	4.0	3.98	99.5
	2.0	1.97	98.5	2.0	1.99	99.5	3.0	2.95	98.3
							2.0	1.99	99.5
Mean ± SD	98.9 ± 0.4			99.1 ± 0.9			99.5 ± 0.6		
*n*	6			6			7		
Variance	0.16			0.81			0.36		
%SE *	0.16			0.36			0.23		
Human urine	5.0	5.00	100.0	6.0	5.96	99.3	7.3	7.30	100.0
	4.3	4.22	98.1	5.3	5.26	99.2	7.0	7.00	100.0
	4.0	3.89	97.3	5.0	4.98	99.6	6.0	5.99	99.8
	3.3	3.28	99.4	4.0	3.99	99.8	5.0	4.98	99.6
	3.0	2.96	98.7	3.0	2.97	99.0	4.0	3.99	99.8
	2.0	1.94	97.0	2.0	1.97	98.5	3.0	2.97	99.0
							2.0	1.99	99.5
Mean ± SD	98.4 ± 1.2			99.2 ± 0.5			99.7 ± 0.3		
*n*	6			6			7		
Variance	1.44			0.25			0.09		
%SE *	0.48			0.20			0.11		

* %SE= SD/$\sqrt{n}.$
